# Untreated dental caries prevalence and impact on the quality of life among 11 to14-year-old Egyptian schoolchildren: a cross-sectional study

**DOI:** 10.1186/s12903-020-01077-8

**Published:** 2020-03-19

**Authors:** Samar Ahmed Eid, Nagwa Mohmmad Ali Khattab, Ahmad Abdel Hamid Elheeny

**Affiliations:** 1 Ministry of Health, Fayum, Egypt; 2 Paediatric and Community Centistry, Faculty of Dentistry, Minia University, Postal code, 61519. Province, Minya. Ard Shalaby, El Minia, Egypt

**Keywords:** Adolescent, Oral health, Dental caries, quality of life

## Abstract

**Background:**

This study aimed to assess caries prevalence and experience among 11 to 14 years, school children, analyze demographic, socioeconomic, personal and professional dental care in relation to untreated carious lesions, and evaluates the effect of decayed teeth on early adolescents’ oral health-related quality of life (OHRQoL).

**Methods:**

A cross-sectional analytical investigation was conducted on 1020 preparatory schoolchildren selected on the basis of a multistage sampling technique. Caries status of the participants detected via recording their caries experience and untreated cavities using DMFT and DT indices. OHRQoL was determined using a validated Arabic CPQ_11–14_ short-form questionnaire. Statistical methods for descriptive analysis, chi-square test, Independent-Samples *t* test and one-way analysis of variance (ANOVA) were used. Multivariate Poisson regression analysis through a hierarchical approach was used to detect the influence of independent variables on DT scores. To declare the association between independent variables and QoL, a step-by-step, multivariate regression analysis was conducted.

**Results:**

The average scores of DMFT and DT in this study were 2.97 ± 1.29 and 1.66 ± 1.24. Poisson regression analysis demonstrated that early adolescents whom their mothers with a lower level of education and of low socioeconomic status were 1.41 and 1.27 times respectively had higher DT scores when compared with their peers. Untreated cavities affected mainly by mother education, school type, family income, and regular dental appointments. Children with DMFT≤3) or DT = 0 recorded a statistically significant lower CPQ_11–14_ average score (p<0.01) and (p<0.0001) respectively.

**Conclusions:**

Untreated carious cavities and caries experience were associated with lower socioeconomic, maternal education andl ess frequent tooth brushing. Untreated carious cavities have a significant negative impact on schoolchildren’s QoL.

## Background

Dental caries is one of the global health issues in both industrialized and developing communities. However, dental caries incidence and prevalence is more pronounced in developing countries like Egypt [1, 2]. Dental caries is an age-related disease involving about 60–90% of schoolchildren [1, 3]. Caries is an outcome of several interplaying factors such as cultural, social, and political factors, which in turn rule the individual behaviors and commitment to preventive oral hygiene measures [4]. Dental caries studying has raised attention in a critical life-stage like adolescence because caries progression increases at this stage [5]. In addition, at adolescence good oral hygiene, proper diet and regular dental check-up behaviors should be enforced. Untreated carious cavities especially when associated with pain may influence children’s’ physical and psychological development as well as school and daily-life achievements [6]. Plenty of studies revealed the association between oral health and adolescent’s quality of life (QoL) physical, psychological, social and emotional aspects [7, 8]. One of the tools designed by Jokovic et al. to measure the oral-health related quality of life (OHRQoL) in children is a child perception questionnaire for 11–14 years age group (CPQ_11–14_) short form [9]. OHRQoL has a multidimensional structure to measure different aspects of oral health in a subjective way including oral symptoms, functional limitations, emotional and functional well-being [10]. Caries prevalence among Egyptian inhabitance is high [1]. The number of investigations conducted in Egypt especially among the early adolescents age group is limited. Moreover, up to the available data, the relation between decayed teeth and OHRQoL among teenagers Egyptian students is still not clarified.

The current study aimed to (1) assess caries prevalence and experience among 11 to14-year-old schoolchildren, (2) analyze demographic, socioeconomic, personal and professional dental care in relation to untreated carious lesions, (3) evaluate the effect of decayed teeth on early adolescent’s OHRQoL.

## Methods

### Design, setting and sampling

**Fig. 1:**
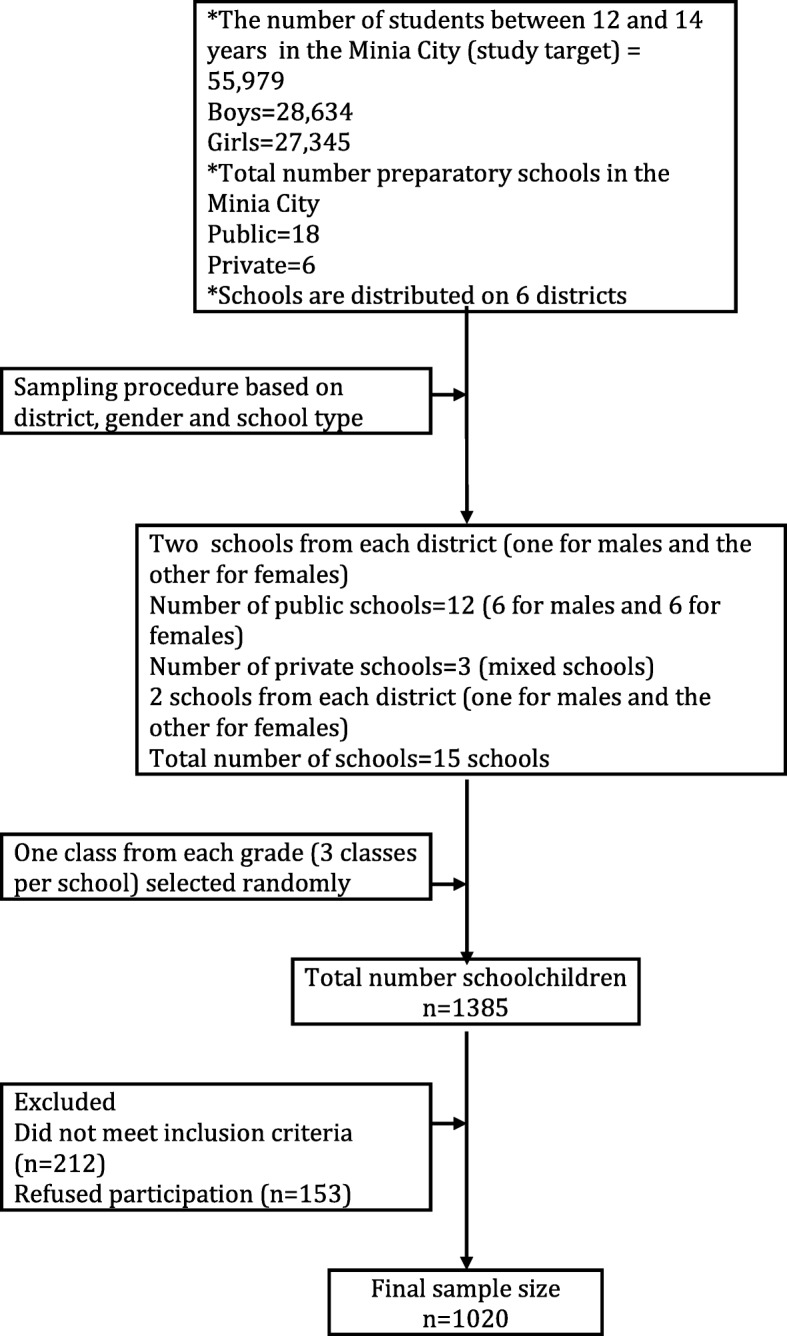
Multistage sampling procedures

A cross-sectional analytical study conducted in Minia City, Upper Egypt between September 2016 and April 2019. The needed number of children participating in the study was 927, which calculated on the basis of the following formula [11]; N = (Z_α/2_)^2^ s^2^/d^2^, where (N) is the number of participants and (d) is the degree of precision adjusted at 0.05 (5%), and the Z_α/2_ was 1.65 A pilot study conducted before launching the procedures on 56 children aged from 12 to 14 years and the standard deviation (s) of DMFT was 0.92. The total sample size was 1020 children after adding 10% to compensate the drop-off. After obtaining the necessary permissions from the Ministry of Education, the sampling process has launched. The type of sample adopted in this study was a multistage stratified random sample. The selected strata based on gender (males and females), school type (public and private schools) and finally the school district (North, South, East or West). Details of sampling procedures illustrated in Fig. 1.

### Eligibility criteria

Children aged from 11 to 14 years should fulfill the following criteria; (1) No orthodontic treatment or malocclusion or severe gingival or periodontal diseases. (2) Absence of any systemic, emotional or intellectual disabilities. (3) No emergency dental recall in the last 3 months.

### Study variables

An anonymous questionnaire using a face-to-face interview has been adopted in this study. The questionnaire was divided into two principal sections; the first section included the following variables; (1) demographic data; (a) gender and (b) age, (2) socioeconomic data (a) mother’s level of education; dichotomized according to the number of education years into ≥9 years (greater than secondary and secondary school) and <9 years (less than secondary school or illiterate), (b) school type; classified into public and private schools, (c) household expenditures which recorded according to the cut-off poverty line in Egypt which is 3.20 US$ per day and [12, 13], (3) dental self-care and use of dental services; (a) frequency of tooth brushing with fluoridated toothpaste, (b) frequency of fluoridated mouth rinse use. The response of both ‘a’ and ‘b’ dichotomized into regular use (frequency is ≥1 time per day) and irregular (no tooth brushing or mouth wash use or ≤ 3 times per week), (c) use of dental floss, (d) Do you regularly visit the dentist for regular check–up per year dichotomized into (yes or no). (4) clinical oral examination and recording dental caries status through recording the Decayed, Missing, Filled Teeth (DMFT) index for permanent teeth according to data into (≤3 and >3) based on the global target of score three announced by World Health Organization (WHO) for the year of 2000 [14]. Untreated carious cavity scores “i.e. Decayed tooth (DT) index” dichotomized into 0 and ≥ 1. The second section concerned with evaluating the OHRQoL using a previously validated Arabic version of CPQ_11–14_ short-form consisted of 16 questions (four questions of each domain). The four domains assessed firstly, the oral symptoms (OS) included questions about pain in teeth/mouth, bad breath, mouth sore and food caught between teeth. The second domain assessed the functional limitation (FL). For instance, the difficulty in chewing of firm food or saying words, sleeping problems and longer time has been taken to eat a meal. The third domain evaluated the emotional well-being (EW) through questions concerned whether the participants felt upset, shy, frustrated or concerned what people think about his/her teeth. Finally, the social well-being (SW) domain illustrated through 4 questions (teased/called names, avoided smiling/laughing, argued with children/family and not wanted to speak/read loud in class). Response scores graduated on a 4-Likert point scale; (0) never, (1) once or twice, (2) sometimes, (3) often and (4) every day or almost every day. The minimum score was 0 and the maximum score was 64. Another one self-perception question reported by each participant about his/her OHRQoL evaluation [9, 15].

### Calibration, pilot study, and data collection

Firstly, two dentists with at least 2 years of residency at the Pediatric and Dental Public Health Department, Faculty of Dentistry, Minia University, trained for 2 weeks for calibration. The second step was conducting a pilot study on 56 children. The pilot study aimed to determine QoL mean and standard deviation required for sample size calculation and to test the intraexaminer and inter-examiner reliability. The results of the pilot study did not include in the final statistical analysis. Dental caries examination performed at two appointments with one-week interval. The clinical examination carried out using a visual-tactile method using a dental mirror and WHO probe 5 s per dental surface under artificial light use [16].

### Statistical methods

**Fig. 2:**
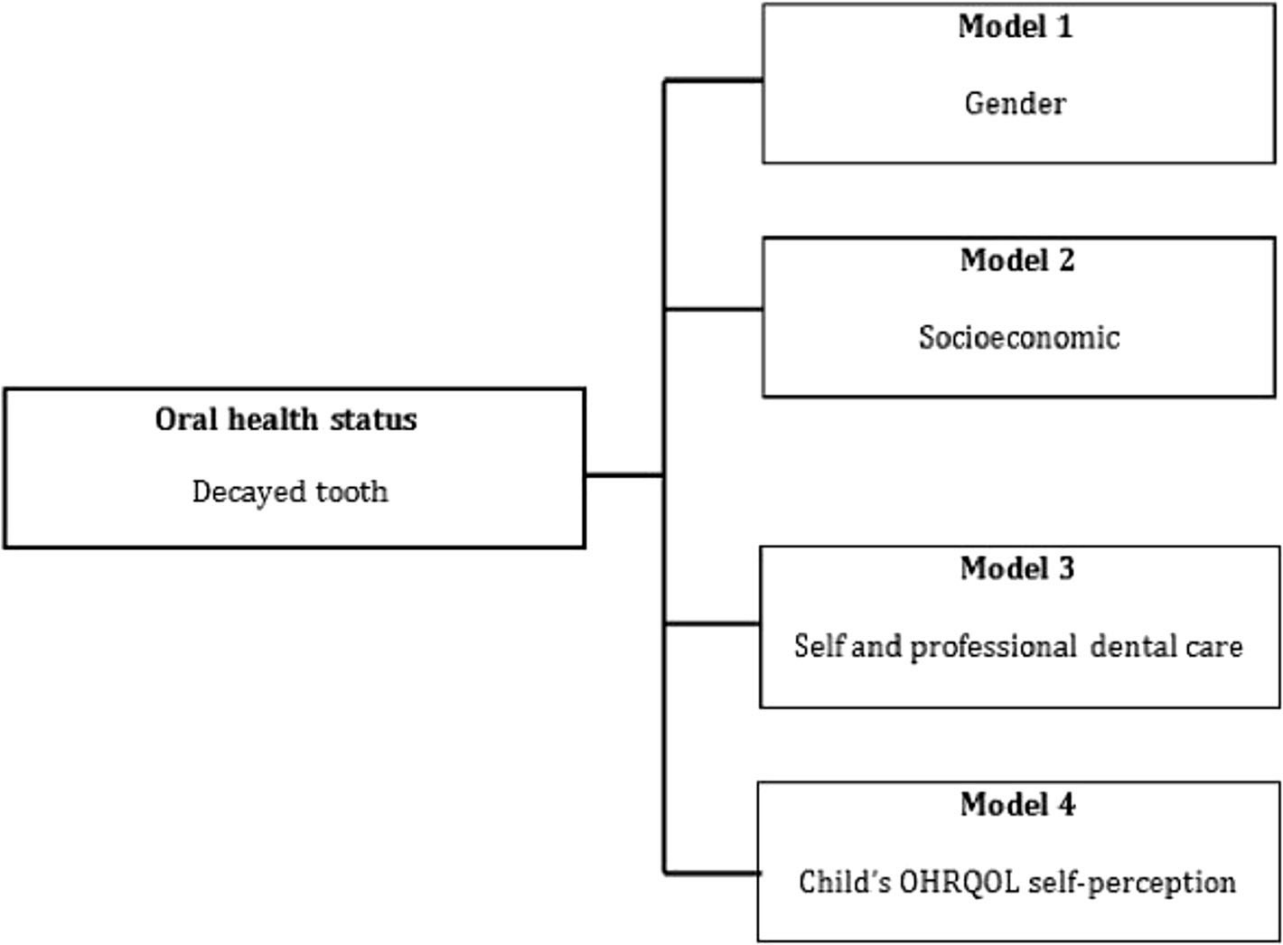
Conceptual model of study variables

A Statistical Program Statistical Package for the Social Sciences (SPSS) version 20 has been used for statistical analysis. Data normality was examined and descriptive analysis including frequency tables, chi-square test for categorical variables, Independent-Samples T-Test and one-way analysis of variance (ANOVA) to compare CPQ_11–14_ overall score means of independent predictors. The mean/standard deviation, and median/Interquartile range (IQR) of the CPQ_11–14_ different domains were calculated. Univariate Poisson regression analysis was performed to determine the associations between decayed teeth (i.e. outcome variable) and demographic, socioeconomic, dental care and oral health related quality of life self-perception independent predictors. Predictors with a significant level exceeding 0.2 (p>0.2) were excluded from the final adjusted multivariate regression model. A conceptual model was released according to a hierarchy approach of determinants and risk factors and it was structured according to the model made by Paula et al. as shown in Fig. 2 [17]. Predictors categorized into four models; Model 1 included gender, Model 2 incorporated model 1 plus socioeconomic variables Model 3 contained Model 2 plus child’s dental care and Model 4 implicated model 3 plus child’s oral health-related quality of life self-perception.

To declare the association between independent variables and QoL, a step-by-step, multivariate linear regression analysis was performed. The best fit defined by the highest R^2^). The level of significance was 5% (p<0.05) and 95% confidence interval (95% CI).

## Results

Intra and inter-observer reliability; Kappa coefficients (κ) were 0.91 and 0.86 for intra-examiner and inter-examiner reliability respectively. More than two-thirds of participants are from the public. The prevalence of dental caries among school children (DT ≥ 1) was 58.5%. Approximately half of the participants showed caries experience exceeded scores three (DMFT>3). The socioeconomic status displayed that more than two thirds of the participants’ mothers had at least 9 years of education (72.1%) and approximately one half the teenager’s family income was below the cut-off point of poverty (46.4%). The majority of the teenagers did not use mouthwash or dental floss (84.8 and 87% respectively). Concerning toot brushing using a fluoridated tooth paste, only 30.1% of the participants were brushing their teeth on a regular basis (2 times daily) (Table 1).

**Table 1:** Frequency of demographic, socioeconomic, dental care, relation caries experience (DMFT) and decayed teeth (DT)

Predictors	N (%)
Gender	
Male	575(56.4)
Female	445(43.6)
Age (years)	
12	323(31.6)
13	299(293)
14	398(39.1)
School type	
Public	721(70.7)
Private	299(29.3)
Mother education (years)	
≥ 9	735(72.1)
<9	285(27.9)
Household expenditure	
>3.20$/day	547(53.6)
≤ 3.20$/day	473(46.4)
Tooth brushing frequency	
Irregular	713(69.9)
Regular	307(30.1)
Mouth washing frequency	
Irregular	865(84.8)
Regular	155(15.2)
Dental appointment per year	
No	671(65.8)
Yes	349(34.2)
Dental floss use	
No	887(87.0)
Yes	133(13.0)
DMFT	
≤ 3	513(50.3)
>3	507(49.7)
DT	
0	423(41.5)
≥ 1	597(58.5)

The average scores of DMFT and DT in this study were 2.97 ± 1.29 and 1.66 ± 1.24. Caries experience showed no statistically significant difference between males and females. Maternal education, school type as an indicator of economic status, regular toothbrush and professional check-up revealed a significant impact on DMFT and DT scores (p<0.001) (Table 2).

**Table 2:** Frequency of demographic, socioeconomic, dental care, in relation caries experience (DMFT) and untreated decayed teeth (DT)

Predictors	DT		p-value*	DMFT		*p-value* *
	DT = 0 N(%)	DT ≥ 1 N(%)		DMFT≤3 N(%)	DMFT>3 N(%)	
Gender						
Male	104(10.2)	471(46.1)	<0.0001	326(32)	249(24.4)	0.53
Female	187(18.3)	258(25.3)		261(25.6)	184(18)	
Age (years)						
12	106(10.4)	217(21.3)	0.06	191(18.7)	132(12.9)	0.29
13	88(8.6)	211(20.7)		179(17.5)	120(11.8)	
14	99(9.7)	299(29.3)		217(21.3)	181(17.7)	
School type						
Public	136(13.3)	585(57.4)	<0.0001	213(20.9)	508(49.8)	0.001
Private	155(15.2)	141(13.8)		179(17.5)	120(11.8)	
Mother education (years)						
≥ 9	423(41.5)	312(30.6)	<0.0001	513(50.3)	182(17.8)	<0.0001
<9	85(8.3)	200(19.6)		170(16.7)	115(11.3)	
Household expenditure						
>3.20$/day	255(25)	292(28.6)	0.01	323(31.7)	224(22)	0.29
≤ 3.20$/day	134(13.1)	339(33.2)		264(25.9)	209(20.5)	
Tooth brushing frequency						
Irregular	125(12.3)	588(57.6)	<0.0001	330(32.4)	383(37.5)	<0.0001
Regular	176(17.3)	131(12.8)		204(20)	103(10.1)	
Mouth washing frequency						
Irregular	268(26.3)	686(67.3)	0.24	358(35.1)	507(49.7)	0.34
Regular	23(2.3)	43(4.2)		93(9.1)	62(6.1)	
Dental appointment per year						
No	136(13.3)	535(52.5)	<0.0001	267(26.2)	404(39.6)	<0.0001
Yes	196(19.2)	153(15)		214(20.1)	135(13.2)	
Dental floss use						
No	260(28.4)	597(58.5)	0.07	372(36.5)	515(50.5)	0.39
Yes	30(2.9)	103(10.1)		72(7.1)	61(5.9)	

*Chi-square test

Poisson regression analysis demonstrates that early adolescents whom their mothers with a lower level of education and of low socioeconomic status were 1.41 and 1.27 times respectively had higher DT scores when compared with their peers. Untreated cavities affected mainly by the mother education, school type, family income and regular dental appointments (Table 3).

**Table 3:** Poisson analysis for decayed teeth (DT) in relation to demographic, socioeconomic and dental care

Predictors	Univariate analysis		Model 1		Model 2		Model 3		Model 4	
	Unadjusted PR 95% CI	*p-value*	Adjusted PR 95% CI	*p-value*	Adjusted PR 95% CI	*p-value*	Adjusted PR 95% CI	*p-value*	Adjusted PR 95% CI	*p-value*
Gender										
Male	1.07(0.98;1.14)	0.17	0.94(0.80;1.11)	0.47	1.07(0.86;1.18)	0.11	0.99(0.84;1.16)	0.90	1.06(0.98;1.14)	0.15
Female	1		1		1		1		1	
Mother education (years)										
<9	1.21(1.06;1.24)	0.002			1.58(1.27;1.95)	<0.0001	1.56(1.25;1.95)	<0.0001	1.41(1.14;1.74)	0.006
≥ 9	1				1		1		1	
School type										
Public	1.14(1.09;1.29)	0.01			1.34(1.09;1.64)	0.006	1.48(1.18;1.85)	0.001	1.27(1.17;1.38)	0.02
Private	1				1		1		1	
Household expenditure										
<3.20$/day	1.16(1.06;3.19)	0.02			1.21(1.03;1.33)	0.018	1.23(1.11;1.39)	0.01	1.22(1.12;1.49)	0.04
≥ 3.20$ /day	1				1		1		1	
Tooth brushing										
Irregular	1.21(1.06;1.49)	0.05					1.02(0.75;1.15)	0.51	1.14(0.80;1.84)	0.46
Regular	1						1		1	
Dental appointments										
No	1.11(1.05;1.23)	0.03					1.20(1.04;1.29)	0.04	1.25(1.16;1.46)	0.004
Yes	1						1		1	
Mouth wash use										
No	1.03(0.79;1.33)	0.85					1.08(0.89;1.94)	0.14	1.18(0.89;1.84)	0.19
Yes	1						1		1	
Dental floss use										
No	1.07(0.70;1.22)	0.30					1.12(0.91;1.28)	0.48	1.05(0.8;1.36)	0.73
Yes	1						1		1	

*PR* Prevalence Ratio

Females report a higher score of CPQ_11–14_ means, indicating lower satisfaction with their OHRQoL than male counterparts (p<0.0001). Regarding caries status, both caries experience (DMFT≤3) and decayed teeth score (DT = 0) record a statistically significant lower CPQ_11–14_ average scores (p<0.01) and (p<0.0001) respectively than those with higher DMFT and DT scores (Table 4).

**Table 4:** Mean of Decayed, Missed, Filled (DMFT) index and decayed teeth (DT) scores in relation to demographic, socioeconomic, dental care, relation

Predictors	CPQ_11–14_ Mean (SD) scores	*p-value* *
Gender		
Male	38.07(12.60)	<0.0001
Female	42.20(10.49)	
Age (years)		
12	40.96(12.14)	0.13
13	42.82(9.64)	
14	41.32(12.66)	
School type		
Public	44.64(8.28)	<0.0001
Private	30.16(12.18)	
Mother education (years)		
≥ 9	40.46(11.67)	0.014
<9	38.13(11.50)	
Household expenditure		
>3.20$/day	36.36(13.26)	<0.0001
≤ 3.20$/day	45.06(6.94)	
Tooth brushing frequency		
Irregular	41.73(11)	<0.0001
Regular	35.33(12.56)	
Mouth washing frequency		
Irregular	40.79(11.46)	<0.0001
Regular	34.77(12.68)	
Dental appointment per year		
No	43.40(9.41)	<0.0001
Yes	34.67(13.23)	
Dental floss use		
No	40.65(11.55)	0.076
Yes	38.73(12.10)	
DMFT		
≤ 3	34.05(12.46)	0.002
>3	42.22(10.14)	
DT		
0	27.66(12.76)	<0.0001
≥ 1	45.48(5.81)	

*Independent-Sample T test; One Way (ANOVA)

OS domain especially, pain figures out the highest mean scores (11.16 ± 3.58) among CPQ_11–14_ different domains. FL, EW, and SW domains average scores are illustrated in Table 5.

**Table 5:** Perceived oral health related quality of life (OHRQoL) based on the scores of child perception questionnaire CPQ_11–14_ domains

CPQ_11–14_ domains	Mean (SD)	Median (IQR)	Cronbach’s alpha
Oral symptoms (OS)	11.16 ± 3.58	12(4.75)	0.71
Functional limitations (FL)	10.45 ± 3.25	11(5)	0.67
Emotional well-being (EW)	10.15 ± 3.42	11(5)	0.61
Social well-being (SW)	9.75 ± 2.98	10(3)	0.67
Overall score	42.40 ± 11.63	43(11.75)	0.84

Multiple regression analysis showed that untreated carious lesions (DT), socioeconomic status and constant toothbrush explain 64% of the variance in participant’s QoL (Table 6).

**Table 6:** Multiple regression analysis of factors influencing oral health related quality of life (OHRQoL) in 11 to 14 years school children

Predictors	β	95% CI	*p-value*	Adjusted R^2^
Step 1				
Gender	0.18	2.75;5.58	<0.0001	0.04
Age (years)	−0.83	−1.67; − 0.26	0.007	
Step 2				
Gender	0.08	0.68;2.98	0.002	0.38
Age (years)	− 0.02	− 0.82;0.31	0.38	
Household expenditure	0.16	2.30;5.01	<0.0001	
School type	−0.54	−15.34;-12.38	<0.0001	
Mother education	−0.23	−8.42;-5.30	<0.0001	
Step 3				
Gender	0.08	075;3.05	0.001	0.4
Age (years)	−0.2	−0.79;0.33	0.43	
Household expenditure	0.14	1.99;4.67	<0.0001	
School type	−0.5	−14.26;-11.04	<0.0001	
Mother education	−0.22	−8.18;-5.04	<0.0001	
Mouth washing frequency	−0.05	−5.24;0.16	0.07	
Tooth brushing frequency	−0.11	−1.59;0.56	<0.0001	
Dental appointment	−0.07	−3.15;-0.21	0.025	
Dental floss use	−0.07	−2.21;-0.17	0.014	
Step 4				
Gender	−0.03	−1.54;0.28	0.17	0.64
Age (years)	−0.03	−0.77;0.09	0.12	
Household expenditure	0.07	0.62;2.70	0.002	
School type	−0.38	−10.97;-8.45	<0.0001	
Mother education	−0.17	−6.36;-3.93	<0.0001	
Mouth washing frequency	−0.04	−3.89;0.28	0.09	
Tooth brushing frequency	−0.07	−1.05;-0.26	0.001	
Dental appointment	−0.08	−1.58;0.73	0.47	
Dental floss use	−0.05	−1.53;1.44	0.95	
DMFT	0.02	−0.34;1.45	0.23	
DT	0.54	12.88;15.03	<0.0001	

## Discussion

Dental caries have several negative impacts, especially on early adolescence life, through reducing the efficiency of masticatory function and general appearance which is reflected in growth and development as it affects emotional and social health. The current observational cross-sectional study intended to assess the caries experience and its impact on OHRQL among a group of Egyptian school children aged from 11 to 14 years. This age group has been chosen to carry out this study based on the following aspects; (1) caries rate is higher among children [18], (2) this age group is the WHO target group for dental caries global comparison [14]. Data were gathered through a face-to-face interviews to eschew data missing, reduce information bias and enhance the accuracy of data [19]. The response rate was 87%, this might be related to the use of anonymous CPQ_11–14_ questionnaire which also guarantees the participant’s confidentiality.

Although the flood of data concerning the worldwide caries experience at various age groups, it still a significant issue to concern. Up to the available data, studies measuring caries experience among 11 to 14-year-old students were limited. Moreover, this work is a leading research in studying the association between caries and OHRQoL among early adolescent Egyptian students. The mean DMFT scores in this study were 2.97 ± 1.29 which approximately similar to the DMFT target declared by the World Health Organization (WHO) in 2000 [14]. Untreated cavities mean values were higher among girls than boys. This difference might be attributed to the higher commitment of girls toward oral hygiene habits like tooth brushing than boys. This finding was in agreement with prior studies [1, 20]. The River Nile is the main source of water in the Egypt. The fluoride contents of Nile waters fluctuate between 0.2 and 0.4 mg-F/L of average of 0.34 mg-F/L. As Minia located in the Upper Egypt where the weather is hot over the year and subsequently the water consumption increases. In addition, the habits of drinking tea and the use of water in cooking are common. Therefore, the final consumption of water which come from the River Nile is approximately 0.7 mg-F/L which compatible with the optimum level of Fluoride announced by The WHO which ranged from 0.7 to 1.2 mg-F/L [21]. Despite of water fluoridation in Egypt provides the optimum level of fluoride, caries prevalence in the current study was high and this might be related to the complex nature of the disease which requires a multidisciplinary approach for its prevention.

In the present study, caries experience was higher than that reported by two former studies conducted in a nearly similar Egyptian age group. The first study performed in 2011, on 976 schoolchildren [2]. The average DMFT score was 1.68 ± 1.92, and this might be assigned to the use of a distinct sampling method which relied on choosing only two public schools (one for boys and the other for girls) from the Giza governorate. In addition, school enrollment of children in Egypt depends fundamentally on geographic distribution. Therefore, the approach might consider children who nearly have similar socioeconomic and parents’ educational backgrounds. The DMFT score of the other study published in 2019, was also lesser than the results of the current study (1.68 ± 1.92) [1]. The reason might be due to the difference between the two studies in sample size. Caries prevalence of untreated carious cavities in the present study was 58.5% which was higher than that reported by a Pontigo-loyola et al. who reported a caries prevalence of 48.6% among 12 and 15 age children in high altitudes [22]. The difference might be related to the higher community water fluoridation which surpassed the systemic water fluoridation in Egypt. The findings of skinner et al. published a caries prevalence of 45% among 14 and 15-year-olds in New South Wales, Australia [23]. In comparison, global DMFT scores reported in other countries showed a wide range of diversity. Some researches revealed lower DMFT scores than the finding of the current research [24–28]. Other research reported nearly similar results [29–31]. While a number of researches recorded higher DMFT scores [14, 32, 33]. This wide range of diversity attributed to the differences in study designs, sample sizes, eating habits and socioeconomic and cultural backgrounds. In the current study, children with lower DMFT and DT scores were belonging to families with higher income as well as education level in comparison to children with high caries experience. This inverse relationship between DMFT scores and both maternal education level and economic status denotes their significant impact on caries experience. Several previous studies confirmed this relationship [34–36]. Lower socioeconomic status might hinder the adherence to preventive interventions such as tooth brushing and regular professional dental examination which consistent with other studies. Moreover, these associations were also evident with untreated carious cavities [37].

Dental caries has a negative impact on the adolescent’s QoL. The present study utilized CPQ_11–14_ short form which reported by Jokovic et al. to have excellent validity and maintained its multidimensional properties [9]. Direct comparison between the results of this study and the findings of global researches concerned with the impact of caries on OHRQoL might be influenced with the diversity of socioeconomic, traditions and cultural backgrounds. However, it was salutary to consider some of these studies. In the present, oral symptoms especially pain demonstrated the highest mean scores which demonstrated the prominent influence on eating, speaking and sleeping aspects. These were in agreement with the findings of former studies [38, 39]. Untreated carious lesions can lead to what could be called a “vicious cycle” because pain or teeth loss as a result of the carious tooth will reduce the mastication performance due to the difficulty in hard food chewing and subsequently, a shift toward more soft and processed food will take place. This food usually more cariogenic and of little nutritional benefits which will increase the caries progression and subsequent increase in more soft food consumption. Another negative aspect of soft and highly processed foods is their passive effect on children’s growth and development [40, 41]. Unfortunately, oral symptoms not the only consequences of pain or missing teeth due to decay in this study, but also, the emotional effect on the participants in this study was announced. This can be explained by the undermining of children’s self-esteem due to altered eating, chewing, speaking and social appearance [41, 42]. The current study emphasized the connection between low socioeconomic status and OHRQoL. The education level of mothers and financial status plays a vital role in an individual’s lifestyle decisions and adherence to routine preventive measures such as tooth brushing and get access to dental services.

The findings of this study spotlight the importance of adopting strategies aimed to increase awareness about oral health among mothers and school children at this age and emphasize the importance of oral hygiene habits. It is also recommended to reduce socioeconomic inequalities.

The present study has a number of strong features such as the representative sample, high response rate as well as intra-examiner and inter-examiner reliability. All of these points are positively reflected in the study generalization as well as its internal validity. On the other hand, the main weak point in this study was attributed to the cross-sectional design as data collected at one point of time in contrast to the prospective design.

## Conclusions

The present study clarifies that more than half of schoolchildren aged from 11 to 14-years showed untreated cavities. This may declare the negative impact of untreated carious lesions and negative QoL. However, caries experience recorded no significant alliance with OHRQoL. Both untreated cavities and caries experience were significantly associated with lower household expenditure, lower maternal education and irregular adherence to tooth brushing habit. Oral symptoms were the chief domains that adversely influencing early adolescent’s OHRQoL. Further studies especially the longitudinal study are required to assess the correlation between these different variables and OHRQoL different aspects.

## Data Availability

The datasets used and/or analysed during the current study are available from the corresponding author on reasonable request.

## Abbreviations

QoL: Quality of Life
OHRQoL: Oral-Health Related Quality of Life
CPQ_11–14_: Child Perception Questionnaire for 11–14 years
DMFT: Decayed, Missing, Filled Teeth
WHO: World Health Organization
DT: Decayed Tooth
OS: Oral Symptoms
FL: Functional limitation
EW: Emotional well-being
SW: Social Well-being
